# Model Specification Searches in Structural Equation Modeling Using Bee Swarm Optimization

**DOI:** 10.1177/00131644231160552

**Published:** 2023-03-29

**Authors:** Ulrich Schroeders, Florian Scharf, Gabriel Olaru

**Affiliations:** 1 University of Kassel, Germany; 2 Tilburg University, Netherlands

**Keywords:** bee swarm optimization, metaheuristics, structural equation modeling, dimensionality, model specification search

## Abstract

Metaheuristics are optimization algorithms that efficiently solve a variety of complex combinatorial problems. In psychological research, metaheuristics have been applied in short-scale construction and model specification search. In the present study, we propose a bee swarm optimization (BSO) algorithm to explore the structure underlying a psychological measurement instrument. The algorithm assigns items to an unknown number of nested factors in a confirmatory bifactor model, while simultaneously selecting items for the final scale. To achieve this, the algorithm follows the biological template of bees’ foraging behavior: Scout bees explore new food sources, whereas onlooker bees search in the vicinity of previously explored, promising food sources. Analogously, scout bees in BSO introduce major changes to a model specification (e.g., adding or removing a specific factor), whereas onlooker bees only make minor changes (e.g., adding an item to a factor or swapping items between specific factors). Through this division of labor in an artificial bee colony, the algorithm aims to strike a balance between two opposing strategies diversification (or exploration) versus intensification (or exploitation). We demonstrate the usefulness of the algorithm to find the underlying structure in two empirical data sets (Holzinger–Swineford and short dark triad questionnaire, SDQ3). Furthermore, we illustrate the influence of relevant hyperparameters such as the number of bees in the hive, the percentage of scouts to onlookers, and the number of top solutions to be followed. Finally, useful applications of the new algorithm are discussed, as well as limitations and possible future research opportunities.

Metaheuristics are general-purpose algorithms that can be used to efficiently solve optimization problems that are computationally too demanding for exhaustive search solutions (Du & Swamy, 2016). Many metaheuristics are inspired by phenomena in nature, which often rely on multiple agents (i.e., population-based approaches). A prominent example is the ant colony optimization (ACO; Deneubourg et al., 1983) algorithm that mimics the foraging behavior of ants. Metaheuristics are adaptive and nondeterministic, as they converge toward optimal or near-optimal solutions by selecting and evaluating over several iterations, using information from previous iterations to adjust the selection process (Colorni et al., 1996; Dorigo & Stützle, 2010). In psychological research, metaheuristics have been used for several purposes, for example, to compile short scales that fulfill certain preset criteria (e.g., Janssen et al., 2017; Leite et al., 2008; G. A. Marcoulides & Drezner, 2001; Olaru et al., 2015, 2019; Schroeders et al., 2016a, 2016b). There is also an extensive research literature on how metaheuristics can be used to correct specification errors between a proposed model and the true model (e.g., G. A. Marcoulides & Drezner, 2003; G. A. Marcoulides et al., 1998; G. A. Marcoulides & Ing, 2012; G. A. Marcoulides & Leite, 2013; G. A. Marcoulides & Schumacker, 2001; K. M. Marcoulides & Falk, 2018). The basic idea behind this model specification search (Long, 1983) is to specify a slightly modified version of an initial, intended model and to compare both in terms of model fit (e.g., Akaike information criteria [AIC] or Bayesian information criteria [BIC]). Manually specifying such competing measurement models is feasible if the model is not too complex and the misspecification not too large, but with an increasing number of variables and possible modifications, this approach is impractical and will cover only a small to negligible part of the parameter space (K. M. Marcoulides & Falk, 2018). Instead, model specification search can be understood as a combinatorial optimization problem (G. A. Marcoulides & Drezner, 2003; G. A. Marcoulides & Schumacker, 2001). For its solution, a variety of metaheuristics (for an overview see G. A. Marcoulides & Ing, 2012) have been proposed such as ACO (Dorigo & Stützle, 2010; Leite et al., 2008), Genetic Algorithm (Holland, 1992; G. A. Marcoulides & Schumacker, 2001), Tabu Search (Drezner & Marcoulides, 1999; Glover, 1986; G. A. Marcoulides et al., 1998), and Simulated Annealing (Černý, 1985; G. A. Marcoulides & Drezner, 1999).

In the present article, we intend to complement previous efforts in model specification search using metaheuristics. Previous approaches and tools are helpful if the researcher has at least a general idea of the structure of the measurement instrument (e.g., number of underlying dimensions). However, in early stages of test or questionnaire development, little is known about the dimensional structure of a measure. Moreover, almost always item selection is necessary because more items are pilot tested than intended for the final instrument. These two requirements—determining the factor structure and item selection—represent a considerable extension of the already challenging combinatorial problem faced in model specification searches with a known number of underlying dimensions. The traditional non-heuristic procedure in psychology is to run an exploratory factor analysis (EFA; Fabrigar & Wegener, 2011) in combination with item selection (e.g., Kruyen et al., 2013; Steger et al., 2022). This approach signifies a number of individual decisions to be made for the structure finding part such as deciding on the estimator (Preacher & MacCallum, 2003), determining the number of factors to be extracted (Preacher et al., 2013), choosing a rotation method (Schmitt & Sass, 2011), and interpreting the solution. Subsequently, items with substantial cross-loadings are excluded and these steps are repeated until a robust and interpretable solution is found. This might seem like a straightforward procedure, but usually it is not. In each iteration of the procedure, there are multiple options. For example, several methods have been suggested to determine how many factors should be retained, but without clear recommendations as to when they should be used (Auerswald & Moshagen, 2019). Thus, item selection with a simultaneous assignment of items to factors is a formidable optimization problem with many researchers’ degrees of freedom (Nelson et al., 2018; Simmons et al., 2011).

To address this issue, we introduce a new population-based metaheuristic to the psychometric literature, a BSO algorithm (for an overview of previous applications in the computational sciences that used algorithms that were motivated by the behavior of bees (see Karaboga & Akay, 2009; Karaboga et al., 2012). In contrast to the foraging behavior of ants that led to the formulation of ACO algorithms, the behavioral repertoire of bees is more sophisticated due to a division of labor. *Scout bees* search for food sources in the surrounding of the hive, whereas *onlooker bees* are directed to certain locations to exploit promising food sources. The main idea of such a division of labor in model specification search is that the global search (scout bees) focuses on the identification of the number of factors and general factor-item allocation, whereas the specific search (onlooker bees) focuses on optimizing a simple structure thereof by reallocating or removing single items. We evaluate the usability and effectiveness of such a BSO in determining the factor structure and selecting items for simple structure in two real data sets representing hierarchical constructs.

## Model Specification Search in Bifactor Modeling

For the current examples, we choose a bifactor model that includes a general factor reflecting the common variance of all items and specific factors covering additional shared variance within subsets of items. The specific or nested factors are orthogonal to each other and to the general factor. Bifactor models have originally been invented in intelligence research (Holzinger & Swineford, 1937), but recently experienced a renaissance as an important structural representation of multidimensionality in various other fields (Reise, 2012; Reise et al., 2010). We chose this model because many psychological constructs are hierarchical in nature (e.g., Kotov et al., 2021; McGrew, 2009; Moshagen et al., 2018) and bifactor models allow for a representation of this hierarchy without imposing proportionality constraints as in higher order models (Brunner et al., 2012). In comparison with a correlated factor model, a bifactor model allows items to load only on the general factor, so that the information of all items can be used, even for items that otherwise cannot be easily allocated to a specific factor.

Consider a measure with n indicators, a strong general factor, and an unknown number of specific factors. The factor model in matrix notation can be denoted as:

y=Λη+ϵ

where η denotes the latent variable, Λ is the factor loading matrix, and ϵ represents the residuals. For instance, the factor model may look as follows:

$$\Lambda =\left[\begin{array}{ccccc} \lambda_{11} & \lambda_{12} & 0 & \cdots & \lambda_{1n}\\ \lambda_{21} & \lambda_{22} & 0 & \cdots & \lambda_{2n}\\ \lambda_{31} & \lambda_{32} & 0 & \cdots & \lambda_{3n}\\ \lambda_{41} & 0 & 0 & \cdots & 0\\ \lambda_{51} & 0 & \lambda_{53} & \cdots & \lambda_{5n}\\ \lambda_{61} & 0 & \lambda_{63} & \cdots & \lambda_{6n}\\ \vdots & \vdots & \vdots & \ddots & \vdots \\ 0 & 0 & 0 & \cdots & 0 \end{array}\right]$$

In this example, the first three indicators load on the general factor (first column) and the first specific factor (second column). The fourth indicator only loads on the general factor, whereas the fifth and sixth indicators load on the general and the second specific factor, and so on. The last indicator is completely excluded as indicated by zero loadings in all columns. The same item-factor assignment that is given in the matrix can also be expressed as a vector of integers where each element represents an indicator and the specific value represents the assignment to a factor (for a similar notation, see G. A. Marcoulides & Drezner, 2001; Murohashi & Toyoda, 2007):

[1 1 1 0 2 2 . . . −1]

Here, positive integers (e.g., 1 and 2 ) denote that an indicator loads on the respective specific factor (e.g., 1 and 2 ) and on the general factor. A value of 0 denotes an item that loads on the general factor only, and −1 represents an item that is excluded from the item pool.

As BSO is a population-based metaheuristic, multiple solutions are followed simultaneously. Each possible item assignment or model is considered a location in the area and a potential food source that is visited by a bee. All models are evaluated by a predefined optimization function including multiple criteria and sorted accordingly (food quality). The better a model is evaluated (promising food source), the more bees are requested to follow and investigate alternative solutions similar to this model—in analogy to the waggle dance of honey bees. In nature, scout bees open up new food sources, whereas onlooker bees search for food in the vicinity of previously explored, promising food sources. With respect to model specification search, scout bees initiate rather global model revisions (e.g., removing a factor), whereas onlooker bees investigate alternative models at a more fine-grained level (e.g., re-assigning a single item). In the following, we will describe the specific operations of the virtual scout and onlooker bees in more detail.

## Main Principles of Bee Swarm Optimization

We identified four key principles of bee foraging behavior and translated them to model specification search:

First, scout bees systematically explore new vicinities based on previously collected information on promising food sources that have been shared in the beehive. We allow scouts to introduce major changes to the best candidate solutions of previous iterations: (a) adding a nested factor (Scout 1 in the following example), (b) splitting a nested factor (Scout 2), (c) removing a nested factor (Scout 3), or (d) merging two nested factors (Scout 4). For instance, these operations could result in the following item assignments:

[1 1 1 0 2 2 2 2 2 0 3 3 3 3 0 4 4 4 4 4 0 4 4 4 −1] (*Best previous solution*)

[1 1 1 5 2 2 2 2 2 5 3 3 3 3 5 4 4 4 4 4 5 4 4 4 −1] (*Scout 1: Add factor*)

[1 1 1 0 2 2 2 2 2 0 3 3 3 3 0 4 4 4 4 4 0 5 5 5−1] (*Scout 2: Split factor*)

[1 1 1 0 2 2 2 2 2 0 0 0 0 0 0 4 4 4 4 4 0 4 4 4 −1] (*Scout 3: Remove factor*)

[1 1 1 0 2 2 2 2 2 0 2 2 2 2 0 4 4 4 4 4 0 4 4 4 −1] (*Scout 4: Merge factor*)

Importantly, each scout *randomly* decides which of the four operations is applied to the best previous solution. If there are multiple possible ways to execute an operation (e.g., which factor could be split), this decision is also made randomly.

Second, onlookers search for food in the immediate proximity of a good food source. In psychometric terms, onlooker bees introduce minor changes to the model that may involve (a) adding an item to a nested factor (if it was not assigned to a nested factor previously, Onlooker 1), (b) removing an item from a nested factor (so that it only loads on the general factor, Onlooker 2), (c) swapping an item between nested factors (Onlooker 3), or (d) completely removing an item from the pool (Onlooker 4). Again, the decision of which these operations are executed and which item is affected is made randomly. For example, minor tweaks may look like these item-factor changes:

[1 1 1 0 2 2 2 2 2 0 3 3 3 3 0 4 4 4 4 4 0 4 4 4 −1] (*Best previous solution*)

[1 1 1 1 2 2 2 2 2 0 3 3 3 3 0 4 4 4 4 4 0 4 4 4 −1] (*Onlooker 1: Adding item*)

[1 1 1 0 0 2 2 2 2 0 3 3 3 3 0 4 4 4 4 4 0 4 4 4 −1] (*Onlooker 2: Removing item*)

[1 1 1 0 2 2 3 2 2 0 3 3 2 3 0 4 4 4 4 4 0 4 4 4 −1] (*Onlooker 3: Swapping items*)

[1 1 1 0 2 2 2 2 2 0 3 3 3 3 0 4 4 4 4 4 0 4 4 −1−1] (*Onlooker 4: Dropping item*)

Third, successful scouts provide information about the location of good food sources (e.g., direction, distance, and quality). The more promising the source, the more onlooker bees are attracted. In psychometric terms, all measurement models are evaluated according to a user-defined optimization function and recorded in a sorted list. In analogy to more profitable food sources leading to intensive dances attracting more onlookers, models that better fulfill the predefined criteria will entail more intense local searches (e.g., 10 onlookers for the best model and eight onlookers for the second best model). Moreover, to streamline the specification search we also included a Tabu list ensuring that the same model is not estimated several times (which may be conceived as depletion of the food source).

Fourth, after each iteration, the list of the best models is updated and used as a starting point by both scout and onlooker bees in the next iteration. To reduce the possibility of focusing too much on only one specific region in the search space (i.e., reaching a local optimum), models that are on top of the best model list are no longer pursued after a predefined number of iterations, which is equivalent to the depletion of food sources. Please note that these models are not removed from the list; they are simply not further explored. Pseudocode for the complete BSO procedure can be found in the online supplement.

## Advantages of Bee Swarm Optimization

BSO has several advantages in test or questionnaire development compared with other methods (see Table 1): First, the algorithm can perform item selection during the optimization. Especially in the early stages of test development, the question of dimensionality underlying a questionnaire is often complicated by the need to select appropriate items from a larger pool. Both exploratory and confirmatory bifactor analyses are not suitable to conduct item selection out-of-the-box. Often items are selected after the structure of the questionnaire was established, for example, by removing items that contributed most to model misfit. However, the dimensionality depends on the specific abbreviated item set, which is why both analysis steps should be conducted simultaneously. Greedy stepwise selection mechanisms also depend on the sequence of item removal; thus, they are prone to run into local optima. In contrast, BSO evaluates item sets during the optimization in a non-greedy manner, that is, it can recover items that would be excluded if only the best solution would have been followed.

**Table 1:** Comparison of BSO Algorithm With Exploratory and Confirmatory Bifactor Modeling.

BSO	Exploratory bifactor	Confirmatory bifactor
Item selection possible	No item selection included	No item selection included
Data-driven	Data-driven	Theory-driven
Simple structure	Cross-loadings	Strong theory needed
	Biased estimates	Anomoulus results
	+ Item selection	
No sequence effect	Sequence effects	
Multiple criteria	One criterion at a time	

*Note.* BSO = Bee swarm optimization. Exploratory/confirmatory bifactor = EFA/CFA with bifactor rotation.

Second, although BSO is logically an exploratory, data-driven approach (Wagenmakers et al., 2012), its models are evaluated using confirmatory factor analyses, allowing to find an unambiguous structure for a measure. The various structure-finding or structure-testing procedures—exploratory factor analysis with bifactor rotation (bifactor EFA), BSO, bifactor exploratory structure equation modeling (BESEM) with target rotation, and confirmatory factor analysis with bifactor rotation (bifactor CFA)—can be ordered on a continuum from exploratory to confirmatory, from no to clear specifications regarding the structure of a measure. Bifactor EFA and BSO are clearly exploratory, whereas bifactor CFA is completely confirmatory. BESEM with target rotation is semi-confirmatory (see Huang et al., 2017), because it combines features of EFA and CFA: On one hand, they allow the occurrence of cross-loadings, and on the other hand, they are also based on a prespecified model structure (Marsh et al., 2010; Morin et al., 2016). BESEM might be useful to take into account the fuzziness of indicators in many real-world settings, but for the target rotation, it is necessary to specify a target structure in advance. More knowledge about a measure will often lead to better models, but in early stages of test development such knowledge is lacking. This is when BSO can prove useful, because the algorithm makes no prior assumptions about the assignment of items to factors and about the number of factors. Nonetheless, BSO enforces a simple structure by only allowing one additional factor loading besides the loading on the general factor, restricting all other loading to zero. This leads to more purified specific factors that might be easier to interpret. However, this clear assignment of items to factors by BSO is not achieved by requiring strong theoretical assumptions as in the case of confirmatory factor analyses.

Third, with respect to item selection, traditional methods rely on stepwise exclusion of items according to a single criterion, for example, deleting the item with the lowest item-total correlation (Kruyen et al., 2013) or the one that contributes most to model misspecification (Whittaker, 2012). Usually, this item deletion is based on a single metric (Clifton, 2020; Steger et al., 2022). In contrast, model search of BSO can incorporate several criteria simultaneously (e.g., model fit, reliability, and length of the scale). The simultaneous inclusion of different optimization goals is a major advantage of metaheuristics in general (Dorigo & Stützle, 2010; Olaru et al., 2015, 2019). For example, metaheuristics have been used to create reliable and cross-culturally invariant short versions of a Big Five personality inventory (Jankowsky et al., 2020). The consideration of different, possibly conflicting criteria in BSO prevents a one-sided optimization at the expense of other criteria (e.g., the attenuation paradox by Loevinger, 1954, and Schroeders et al., 2016b).

Fourth, bifactor models might run into estimation problems or produce anomalous results. In case of exploratory bifactor modeling, Schmid–Leiman and alternative bifactor rotations lead to inaccurate parameter estimates when the specified model departs from perfect cluster structure (e.g., item cross-loadings on nested factors; Mansolf & Reise, 2016). In case of confirmatory bifactor modeling, overfactoring might lead to anomalous results. Therefore, Eid et al. (2017) proposed two theory-driven alternative models, the bifactor-(S–1) model and the bifactor-(S.I–1) model, in which either a factor or a single item per factor is set as a reference. In case of clear theoretical assumptions, such reference models are the preferred choice, but without a clear understanding of the factor structure, these models are difficult to specify. In contrast to these bifactor models with a reference, BSO makes no prior assumption how items should be assigned to factors, which is completely subjected to the optimization process. Thus, BSO can circumvent the issue of overfactoring by allowing items to load on the general factor only.

Fifth, in comparison with an exhaustive search, BSO is efficient. The combination of structure finding and item selection is a huge combinatorial problem (G. A. Marcoulides & Drezner, 2001). To give an idea on the combinatorial complexity, let us assume a bifactor model with 25 items, and, for the sake of simplicity, we restrict the number of nested factors to a maximum of two. Each item can thus take one of four states (item is excluded, loads on the general factor only, loads on either the first or second nested factor), which results in a total of approximately $4^{25}=1,125,899,906,842,624$ models (if models with less than three items per factor are counted). If we estimate that each model takes one-tenth of a second to compute, then even with parallel computation on 10 cores, the analysis of all models would take 356,776 years. In comparison, BSO achieves results in considerably less time in similar settings (as in the following empirical examples)—usually between 1 and 3 hr.

## Research Objectives

The aims of the present proof-of-principle study are twofold: First, we will systematically study the influence of different hyperparameters such as the number of scout bees and onlooker bees, respectively, on the convergence and the quality of the provided solutions. For this purpose, we used the well-known Holzinger–Swineford data set to specify a bifactor model with all subtests loading on a general factor and in addition on two to five domain-specific, nested factors. The task of the BSO algorithm is to group the indicators into nested factors where the number of nested factors is not specified further. The optimization follows a predefined function that takes into account different criteria such as model fit, minimal factor loading, and the total number of items. The results will be compared with the results of exploratory and confirmatory factor analyses with Schmid–Leiman rotation and a varying number of nested factors.

Second, we study the usability and applicability of BSO in another data set featuring a (SD3, Jones & Paulhus, 2014). In the last decade, personality traits that were associated with ethically, morally, and socially questionable behavior have received a lot of attention among personality researchers (e.g., Moshagen et al., 2018). The SD3 consists of 27 items with nine items each for machiavellianism (a manipulative attitude), narcissism (excessive self-love), and psychopathy (lack of empathy). Because the factor structure of the SD3 has not been satisfactorily clarified (Jones & Paulhus, 2014; Persson et al., 2019), we use the questionnaire as an example to showcase the possibilities of the BSO algorithm. More precisely, we demonstrate how to derive essentially equivalent models that all satisfy the predefined psychometric criteria, thus, both streamlining the item pool of a measure and exploring its structure.

## Method

### Data sets

#### Holzinger–Swineford Data Set

We used the Holzinger–Swineford data set, which is a classic textbook example that has been reanalyzed many times in the psychometric literature (e.g., Murohashi & Toyoda, 2007). The data set contains intelligence test scores for 24 cognitive tests from 301 children attending two schools (for more information, see Holzinger & Swineford, 1939). The study on which the data are based has become famous in the assessment literature because it was the first application of a bifactor model (Holzinger & Swineford, 1937).

Concerning the structure of the Holzinger–Swineford data set, the 24 subtests were chosen from five different broad abilities (number of subtests in parentheses): (a) spatial abilities (4), (b) verbal abilities (5), (c) mental speed (4), (d) memory (6), and (e) mathematical deduction (5). Instead of the intended factor structure, Holzinger and Swineford (1939) concluded that a bifactor model with four specific factors (without the last factor) better fitted the data.

#### Dark Triad Data Set

One of the most influential questionnaires to measure the dark core of personality is the SD3, in which previously separate developmental strands are merged by combining and abbreviating existing measures (Jones & Paulhus, 2014). Data were collected online and distributed under an Open Database License.^1^ The data set consists of the responses of 18,192 participants on the 27 items of the SD3.

With respect to the structure of the measure, the questionnaire developers described the fit of the three-dimensional model in the original publication as “not impressive (RMSEA [Root Mean Square Error of Approximation] = .07, CFI [Comparative Fit Index] = .82, and TLI [Tucker-Lewis-Index] = .80)” (Jones & Paulhus, 2014, p. 33). Consequently, Persson et al. (2019) investigated the psychometric properties of the SD3 in three independent samples and proposed a bifactor structure with an overarching dark personality as general trait and either three nested factors or two nested factors, in which machiavellianism and psychopathy collapse, while narcissism is modeled as a second independent factor.

### Hyperparameters of the Bee Swarm Optimization Algorithm

Metaheuristics often provide efficient solutions to a problem, but not necessarily the best solution. The convergence behavior of the BSO depends on the optimization function and several hyperparameters regulating the search behavior and restricting the search space (the most important are given in Table 2). To study their influence in more detail, we systematically varied the following three hyperparameters in the Holzinger–Swineford data set: (a) the number of bees in the hive (n_bees), (b) the percentage of scouts among all bees (percent_scouts), and (c) the number of top solutions that attract onlooker bees (top_best). n_bees should have a direct influence on the convergence behavior and on the duration of the search: The more bees, the larger the area that can be covered and the more extensive the search. The other two hyperparameters (percent_scouts and top_best) affect the relation of diversification to intensification. The higher the proportion of scouts among all bees, the more diversification occurs compared with intensification. The top_best parameter determines how many promising solutions are intensified. A small number indicate a strong intensification but also a more specific local search.

**Table 2:** Hyperparameters of the BSO Algorithm,

Argument	Explanation	Example
n_bees	Number of bees in the hive	200
percent_scouts	Proportion of scout bees	.25
top_best	Number of top solutions to be examined	20
max_iter	Maximum number of iterations without improvement	10
min_nest_fac	Minimum number of (nested) factors	1
max_nest_fac	Maximum number of (nested) factors	5
depletion	Number of iterations after which the food source is depleted	5

*Note.* Arguments that are varied in the empirical illustration are given in the upper part of the table.

We chose the Holzinger–Swineford data set for this demonstration because researchers have a good understanding of the structure (e.g., Murohashi & Toyoda, 2007). In more detail, we used the following design for the empirical illustration of the hyperparameters: n_bees with six levels (100, 200, 400, 600, 800, and 1000), percent_scouts with three levels (.25, .50, and .75), and top_best with three levels (10%, 20%, and 50% of n_bees). Each combination was run across 20 seeds, totaling 6·3·3·20=1080 runs. Because the overall best solution is essentially unknown, we considered the overall best solution across all runs according to our optimization criteria the best solution.

### Statistical Analysis

The BSO algorithm is openly available from Github: https://github.com/FlorianScharf/BSO. BSO relies mainly on the parallel package for parallel computing (v.4.2.2; R Core Team, 2022), lavaan for CFA (v.0.6.13; Rosseel, 2012), and ggplot2 for plotting the convergence plots (v.3.4.0; Wickham, 2016). All syntax and data files necessary to reproduce the analyses of this study can be found in an online repository: https://osf.io/kx4fg/.

### The Optimization Function

The core of a metaheuristic search is the optimization function that evaluates the quality of the models. The optimization function is modular in design and often contains several criteria. In the present context, we focused on model fit, factor loadings, and the number of items included in the final model. All criteria were logit-transformed to scale the outcomes of the different formulae (e.g., Equations 1 and 2) to the range between 0 and 1 to maximally differentiate between models around a preset cutoff value (Janssen et al., 2017; Schroeders et al., 2016a). We labeled these value ν in reference to the Greek word nectar ( νϵκταρ ).

With respect to the first criterion, model fit, we used a combination of an incremental fit index, the Comparative Fit Index (CFI ≥.95), and an absolute fit index, the Root Mean Square Error of Approximation (RMSEA ≤.06)—as proposed with the two-index presentation strategy (Hu & Bentler, 1999). All models had continuous indicators, which is why the maximum likelihood estimator was used. To streamline the convergence of the BSO algorithms by reducing the number of non-promising models, both observed and latent variances were forced to be non-negative (bounds = “pos.var”). The chosen cutoff values for the fit indices constitute the inflection point of the function.

(1) $$\nu_{\mathrm{CFI}}=\frac{1}{1+\exp(50\cdot (.95-\mathrm{CFI}))}$$

(2) $$\nu_{\mathrm{RMSEA}}=\frac{1}{1+\exp(-50\cdot (.06-\mathrm{RMSEA}))}$$

The second criterion of the optimization function deals with the minimal absolute factor loading across all nested factors, which was set to λ=.30 .

(3) $$\nu_{\lambda }=\frac{1}{1+\exp(15\cdot (.30-|\lambda_{\min}|))}$$

The third criterion deals with the number of indicators that will be used in the final version. The indicators in both data sets had been extensively tested; that is, item selection had already taken place in previous stages of test development. For example, the original version of SD3 consisted of 41 items from which the final 27 items were drawn. For this reason, we choose a cutoff of 95% of all items, which essentially prevents any substantial item selection.

(4) $$\nu_{\mathrm{items}}=\frac{1}{1+\exp(30\cdot (.95-n(\mathrm{items})))}$$

Please note that the chosen cutoffs slightly differ in the second example to accommodate for the different data set, that is, for the dark triad data set we deemed a CFI ≥.90 satisfactory and strived for a minimal absolute factor loading ≥.20. The overall weighting of the three criteria in the optimization function favors the model fit:

(5) $$\nu_{\mathrm{total}}=2\cdot (\nu_{\mathrm{CFI}}+\nu_{\mathrm{RMSEA}})+\nu_{\lambda }+2\cdot \nu_{\mathrm{items}}$$

Models in which one of the criteria was below a predefined threshold of ν≤.0001 were not followed up. Please note that other criteria could also easily be implemented in the optimization function such as maximizing correlations with covariates, favoring item sets that offer measurement invariance with respect to grouping variables, balancing content domains, etc. In this introduction to BSO, we will adhere to some basic principles of test construction.

## Results

### Example 1: Holzinger–Swineford Data Set

Specifying the originally intended bifactor model with five nested factors (Holzinger & Swineford, 1939) resulted in a confirmatory factor model with acceptable fit: χ^2^(228, *N* = 301) = 438.9, *p* < .001, CFI = .922, RMSEA = .055. However, the standardized loadings of the fifth factor (deduction) fluctuated unsystematically around 0, indicating overfactoring. A confirmatory bifactor model with four specific factors and the indicators of deduction set as reference overcame these problems: χ^2^(233, *N* = 301) = 444.0, *p* < .001, CFI = .922, RMSEA = .055. With respect to the optimization function which was the base for the BSO algorithm, this model yielded a ν -value of 3.19.

As another point of comparison, we conducted exploratory factor analyses and Schmid–Leiman rotation with two, three, and four specific factors (i.e., SL2, SL3, and SL4). In a subsequent step, we assigned all items to the general factor only that had a factor loading below .30 on the nested factor to make a reasonable comparison with the BSO. The bifactor solution with two nested versus three nested factors came to similar values in the optimization function ( $\nu_{2}$ = 2.65 vs. $\nu_{3}$ = 2.65), whereas the solution with four nested factors scored higher ( $\nu_{4}$ = 3.42).

The best model derived by the BSO algorithm was a bifactor model and four specific factors, resulting in a substantially higher value in the optimization function ( ν = 3.88). Compared with the intended structure, there were some modifications: One indicator of the memory factor (*figurew*) only loaded on the general factor and two indicators that were originally intended for the last factor (*numeric* and *arithmet*) loaded on the mental speed factor instead. This last modification seems reasonable because speed played a crucial role in solving these simple arithmetic problems. The model fit was slightly better: χ^2^(232, *N* = 301) = 414.6, *p* < .001, CFI = .933, RMSEA = .051 (parameter estimates for the four-dimensional solutions are given in the online supplement). Given the constraints specified in the optimization function, this solution was deemed the optimal solution in the context of the subsequent illustration.

**Figure 1:**
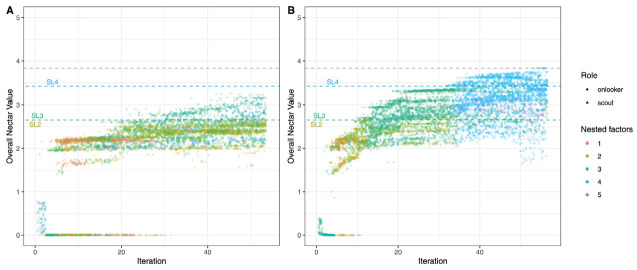
Convergence Plots of Two Exemplary BSO Runs. *Note.* In both runs, the same optimization function was used, but with different hyperparameters. Left panel: n_bees = 120, top_best = 60, percent_scouts = .75, Right panel: n_bees = 120, top_best = 24, and percent_scouts = .25. Colored dashed lines marked with SL2, SL3, and SL4 represent the nectar values of the confirmatory bifactor model with two, three, and four specific factors and Schmid–Leiman (SL) rotation. Nectar values of SL2 and SL3 are almost identical. The gray dashed line indicates the empirical optimum. The syntax can be found online at https://osf.io/jvrpq.

Figure 1 shows the convergence plots of two exemplary specifications of the BSO algorithm with the same optimization function and a total of 120 bees. In the left panel, the top 60 solutions were pursued in each iteration, whereas only the best 24 solutions were used in the right panel. In addition, the percentage of scouts differed across the two examples: 50% versus 25%. As a consequence, the process in the left panel was less diversified and ran into a local minimum, while the optimum solution was found in the right panel. This led to the question which hyperparameter settings have a positive influence on the convergence behavior.

### Systematic Variation of Hyperparameters

**Figure 2:**
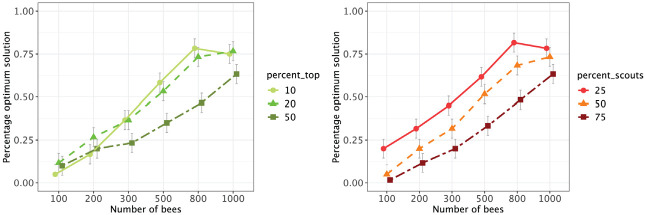
Proportion of Optimal Solutions Among Random Starts as a Function of Hyperparameters. *Note.* Points represent the average proportion of optimal solutions across all random seeds per condition, and error bars represent ±1 standard error.

Overall, the algorithm provided consistent and efficient results in the Holzinger–Swineford data set, because even with a small number of bees the optimal solution was often found across 20 seeds. With 800 or more bees, the optimal solution was provided in almost all cases (for more detailed results, see online supplement). To determine the influence of the hyperparameters on the consistency of the results, we conducted a three-way analysis of variance (ANOVA) with the hyperparameters as independent variables and the frequency of how often the best solution was found across 20 seeds as the dependent variable. The main effects of all three hyperparameters were significant, with the number of bees being the most influential factor, $F(5,1026)=63.99,p<.001,\eta_{p}^{2}=.24$ . Figure 2 indicated improved consistency of results if only 10% or 20% of the best models were followed up in subsequent iterations, $F(2,1026)=10.96,p<.001,\eta_{p}^{2}=.02$ . Moreover, using only 25% scouts (and 75% onlookers) yielded better results than the opposite ratio, $F(2,1026)=27.78,p<.001,\eta_{p}^{2}=.05$ . In summary, at least for this example, it is important for the BSO to weigh intensification more strongly than diversification. Also, the interaction between the percentage of scouts and the number of top solutions to be followed was significant (for the complete results of the ANOVA, please see online supplement).

### Example 2: Dark Triad Data Set

The intended confirmatory bifactor model with nine items loading on each of the three specific factors machiavellianism, psychopathy, and narcissism (Jones & Paulhus, 2014) had sufficient model fit, χ^2^(297, *N* = 18,192) = 17,175.1, *p* < .001, CFI = .901, RMSEA = .057. However, some of the factor loadings on the nested factors turned out to be low, that is, three were below .10 and eight below .20. The factor saturation of the nested factors was low for machiavellianism ( $\omega_{M}=.11$ ) and for psychopathy ( $\omega_{M}=.19$ ), and substantial for narcissism ( $\omega_{M}=.44$ ). The ν -value of this theoretically established model was ν = 3.87.

Table 3 gives the best solutions of 10 independent seeds, out of which seven yielded higher ν -values than the intended model. The best model had essentially the same model fit as the theoretical model, but a slightly higher minimal factor loading on the nested factors ( $\lambda_{\min}$ = .108 vs. $\lambda_{\min}$ = .074; for all factor loadings of the best BSO model, see online supplement). The right side of the table shows the color-coded item-factor assignment. Overall, the structure found by the BSO algorithm matches the intended structure. There were two deviations of the best solution from the intended structure: First, two machiavellianism items loaded on the general factor only (see light gray squares). Second, the item *“Whatever it takes, you must get the important people on your side”* was assigned to narcissism instead of machiavellianism (with the item arguably measuring grandiosity). Both modifications can be understood as a sign that the specificity of the machiavellianism items was lower.

**Table 3:** The Best BSO Solutions of 10 Seeds Concerning the Structure of the SD3.

Seed	ν	CFI	RMSEA	$\lambda_{\min}$	Item-factor assignment	
9	3.968	.902	.056	.108	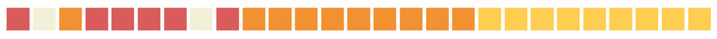
1	3.935	.902	.056	.092	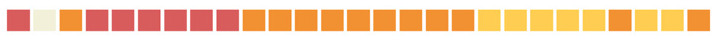
4	3.935	.902	.056	.092	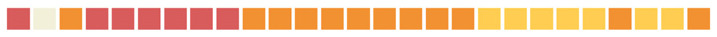
5	3.935	.902	.056	.092	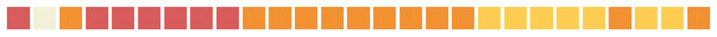
7	3.935	.902	.056	.092	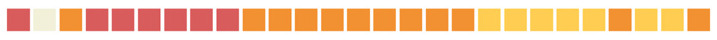
8	3.935	.902	.056	.092	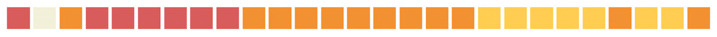
2	3.907	.902	.056	.078	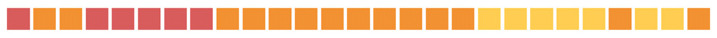
6	3.698	.893	.058	.106	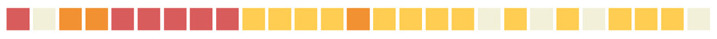
10	3.637	.890	.059	.115	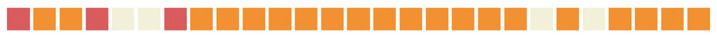
3	3.633	.892	.059	.097	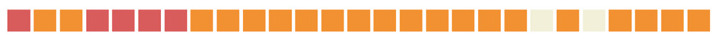
					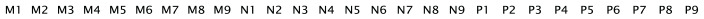

*Note.* In all runs, the same optimization function was used with CFI ≥.90, RMSEA ≤.06, | $\lambda_{\min}|\geq .20$ , and item selection essentially turned off. Hyperparameters: n_bees = 2000, top_best = 200, percent_scouts = .25. M1-M9 = machiavellianism; N1-N9 = narcissism; P1-P9 = psychopathy; red/orange/yellow = nested factors; light gray = general factor only.

## Discussion

In this article, we introduced a new metaheuristic—BSO—to the psychometric literature. In the course of measurement development, behavioral researchers often have to make several decisions, such as determining the number of factors to extract, selecting items, and thinking about alternative assignments of items to factors—rendering the process a formidable optimization problem. BSO tackles the question of dimensionality of a measurement instrument in combination with a possible item selection. Both structure finding and the item selection are conducted jointly according to an optimization function that may include several criteria (e.g., model fit and factor saturation). In contrast to exploratory bifactor analysis, BSO avoids biased estimates (Mansolf & Reise, 2017) and renounces cross-loadings that sometimes complicate the interpretation of factors. In contrast to confirmatory bifactor analysis, BSO does not presuppose strong theoretical assumptions and avoids the sometimes occurring anomalous results (Eid et al., 2017). With regard to the extensive metaheuristic literature concerning model specification search (G. A. Marcoulides & Drezner, 2003; G. A. Marcoulides et al., 1998; G. A. Marcoulides & Ing, 2012; G. A. Marcoulides & Leite, 2013; G. A. Marcoulides & Schumacker, 2001; K. M. Marcoulides & Falk, 2018), both similarities and differences to BSO can be found. One obvious commonality is that metaheuristics are often based on biological mechanisms of an animal population (e.g., a fish swarm or an insect colony). In addition, many metaheuristics try to strike a balance between two opposing strategies—diversification (or exploration) versus intensification (or exploitation)—and they will be more successful in finding a near-to-optimal solution to a given optimization problem if a (dynamic) balance between both strategies can be established (Blum & Roli, 2003). For the model specification search discussed in the current article, measurement and structural parameters of a structure equation modeling (e.g., factor loadings and factor correlations) are conceptualized as a vector that is subject to a random modification (see G. A. Marcoulides & Drezner, 2003; G. A. Marcoulides & Schumacker, 2001). The resulting model is then evaluated regarding predefined criteria which in subsequent iterations influences the probability of items to be assigned to factors. Even though the algorithms are similar on this abstract level and often achieve similar results, there are still major differences between the various algorithms (G. A. Marcoulides & Ing, 2012; Olaru et al., 2015; Schroeders et al., 2016b). If one wants to draw parallels between ACO and BSO, one could link the local search of the onlooker bees to the mode of operation of the ants, whereas the global search of the scout bees has no direct equivalent. In ACO, exploration and exploitation depend on the number of ants, search duration, and evaporation of pheromones. In BSO, however, the two processes are split with scouts engaging in diversification and onlookers in intensification. Such a division of labor might favor a dynamic balance between both strategies when it comes to finding a measure’s structure. Compared with metaheuristics that have previously been used, BSO brings two major features: First, the number of factors does not have to be specified a priori, and second, the search for a solution can be combined with item selection. Thus, we deem BSO a flexible and promising new tool in the method toolbox of psychometricians.

The measurement model that is finally presented in an article is often equivalent in terms of its psychometric properties (e.g., model fit and factor saturation) to other models. Such alternative models might offer substantively meaningful concurrent explanations to the data (MacCallum et al., 1993). In practice, however, the number of models to be manually specified and tested is reduced to a small subset of models that are theoretically motivated. If a researcher tries to specify all conceivable models, an iterative, knowledge-driven search is neither practical nor exhaustive (G. A. Marcoulides & Ing, 2012; K. M. Marcoulides & Falk, 2018). BSO offers a different solution to this problem: BSO systematically searches the solution space of competing measurement models, so that researchers get a sense of the variation of possible good (not essentially equivalent) solutions. In this sense, BSO is a good complement to more theory-driven methods to assess the robustness of effects such as specification curve analysis (Simonsohn et al., 2020) or multiverse analysis (Steegen et al., 2016). In contrast, the BSO algorithm provides the researcher with a set of appropriate, but data-driven models that need to be cross-validated before claiming real validity (G. A. Marcoulides & Leite, 2013). Based on this set, alternative models can be evaluated with respect to their usefulness and theoretical plausibility. As exemplified with the SD3 data set, the BSO can thus point to weaknesses in test development (i.e., low specificity of the machiavellianism items).

The present article is a first-time proof of principle, which implies several limitations but also possible future research avenues. First, our presentation of BSO is currently limited to a bifactor model; however, it should be easy to extend the algorithm to other models such as a correlated factor model or a higher order model. The general idea of scout bees which introduce major changes to the model specification search and onlooker bees which engage in minor tweaks could be useful for several models. Second, we deliberately decided to leave the addition/removal of factors (or items to factors) to chance. From the convergence behavior in the examples, we conclude that the algorithm efficiently leads to a stable final solution within a few dozen iterations. Conversely, the specification search could also be more restrictive or goal-oriented, that is, the algorithm presumably can be further optimized at the expense of losing the connection to the biological model. For example, items could be added/removed with Tabu search (for a successful combination of ACO and Tabu search, see Jing et al., 2022) or the items subject to change could be selected based on their contribution to the model misfit (e.g., modification indices). Third, we tried to closely mirror the foraging behavior of honey bees, but also made simplifications to the rather complex communication of bees. Thus, one might question specific decisions we made in translating the biological principles into psychometric concepts. We acknowledge that the chosen approach is not the only conceivable implementation of a BSO algorithm. But just as there are different types of bees (with a diverse behavioral repertoire), there are also different BSO algorithms that could be developed (for a critical discussion of the use of metaphors in the development of new methods, we recommend Sörensen, 2015). The general idea of scout bees that introduce major changes to the model specification search and onlooker bees that engage in minor tweaks could also be useful for a wide range of applications. At this point of algorithmic development, we also know little about whether the presented set of results on hyperparameters generalizes to other data sets. Presumably, the degree to which intensification or diversification leads to better performance of BSO depends on the nature of the solution space and the optimization function. We found that 25% scouts led to better results than a higher percentage of scouts, which is interestingly backed up by observations of honeybees for which the number of scouts varies between about 5% and 35%, depending on forage availability (Seeley, 1983). To answer such questions concerning the hyperparameter more systematically, it is necessary to conduct a simulation study with different optimization problems, which is beyond the scope of this introduction.

On a more general methodological stance, we think we have just tapped the potential of metaheuristics in psychometrics. Similar optimization problems are widespread: For example, constructing parallel test forms or booklets with many boundary conditions (e.g., average item difficulty, comparable test information curves, and predefined item coverage) is a common and reoccurring issue in educational large-scale assessment and represents another optimization problem that could also be handled with metaheuristics (see van der Linden, 2005). In addition, current applications of metaheuristics often evolve around the items of a measure (e.g., constructing short scales), but it is also possible to apply the same logic to the person side, that is, metaheuristics could be used to form groups of persons (rather than item bundles). In short, potential new applications in psychology are manifold, as well as the possibilities for further methodological developments. Therefore, we welcome the recent efforts and advances of other researchers to capitalize on the possibilities of metaheuristics such as combining ACO and Tabu search for model specification (Jing et al., 2022) or using ACO to automatically search for sensitivity parameters (Leite et al., 2022).

## Supplemental Material

sj-pdf-1-epm-10.1177_00131644231160552 – Supplemental material for Model Specification Searches in Structural Equation Modeling Using Bee Swarm Optimization Click here for additional data file. Supplemental material, sj-pdf-1-epm-10.1177_00131644231160552 for Model Specification Searches in Structural Equation Modeling Using Bee Swarm Optimization by Ulrich Schroeders, Florian Scharf and Gabriel Olaru in Educational and Psychological Measurement

## Notes

1.
https://www.kaggle.com/datasets/lucasgreenwell/short-dark-triad-responses
