# Supplementary material for: Model Specification Searches in Structural Equation Modeling Using Bee Swarm Optimization
Source: Educ Psychol Meas. 2023 Mar 29;84(1):40–61. doi: 10.1177/00131644231160552 (PMC10795566; doi:10.1177/00131644231160552)
Supplement: sj-pdf-1-epm-10.1177_00131644231160552 – Supplemental material for Model Specification Searches in Structural Equation Modeling Using Bee Swarm Optimization [file sj-pdf-1-epm-10.1177_00131644231160552.pdf]

Online Supplement: Model Specification Searches in Structural Equation Modeling Using Bee Swarm Optimization

List of Figures

1 Pseudo code for the Bee Swarm Optimization (BSO) Algorithm 2

List of Tables

1 Results of Systematic Variation of BSO Hyperparameters 3
2 ANOVA for the Results of the Hyperparameter Variation 5
3 Standardized factor loadings of the best BSO solution for the HS data set . . 6
4 Standardized factor loadings of the best BSO solution for the SD3 data set . 7

Figure 1

Bee Swarm Optimization (BSO)

```
for (iteration = 1 : max_iteration) do
    if (iteration == 1) then                                  ▷ 1st iteration
        for (1 : bees) do
            Randomly compile models
            fit = evaluate(model)
            best_solutions = Take best models
    else
        while (count < max_iteration) do
            Continue if flowers are not depleted
            Split bees into scouts and onlookers
            for (1 : bees) do
                Use best_solutions as starting point
                if (bee == scout) then                        ▷ Scout bees
                    Select one of the following options:
                      Condition 1: Add factor
                      Condition 2: Split factor
                      Condition 3: Remove factor
                      Condition 4: Merge factors
                else                                          ▷ Onlooker bees
                    Use best_solutions as starting point
                    Select one of the following options:
                      Condition 1: Add item to nested factor
                      Condition 2: Remove item from nested factor
                      Condition 3: Swap item between nested factors
                      Condition 4: Delete item completely
            fit = evaluate(model)
            if (fit > best_solution) then
                Update the best solution
            else
                depletion + 1
        count + 1
```

Table 1
Results of Systematic Variation of BSO Hyperparameters

Bees	Scouts	Top	Min	M	Max	SD	Percent
100	0.25	0.1	0.000	2.496	3.833	1.695	0.15
100	0.25	0.2	0.000	2.766	3.833	1.647	0.25
100	0.25	0.5	0.000	2.975	3.833	1.528	0.20
100	0.50	0.1	0.000	2.363	3.705	1.780	0.00
100	0.50	0.2	0.000	3.154	3.833	1.361	0.10
100	0.50	0.5	0.000	3.094	3.833	1.343	0.05
100	0.75	0.1	0.000	2.389	3.705	1.634	0.00
100	0.75	0.2	0.000	2.884	3.705	1.486	0.00
100	0.75	0.5	2.882	3.475	3.833	0.294	0.05
200	0.25	0.1	0.000	2.774	3.833	1.650	0.20
200	0.25	0.2	0.000	2.991	3.833	1.543	0.45
200	0.25	0.5	0.000	3.368	3.833	1.154	0.30
200	0.50	0.1	0.000	2.959	3.833	1.526	0.20
200	0.50	0.2	0.000	3.107	3.833	1.356	0.25
200	0.50	0.5	0.000	3.407	3.833	0.840	0.15
200	0.75	0.1	0.000	2.849	3.833	1.482	0.10
200	0.75	0.2	0.000	3.045	3.833	1.332	0.10
200	0.75	0.5	0.000	3.376	3.833	0.834	0.15
300	0.25	0.1	0.000	2.947	3.833	1.527	0.35
300	0.25	0.2	0.000	3.416	3.833	1.171	0.70
300	0.25	0.5	0.000	3.342	3.833	1.154	0.30
300	0.50	0.1	0.000	3.139	3.833	1.370	0.40
300	0.50	0.2	0.000	3.282	3.833	1.150	0.35
300	0.50	0.5	3.514	3.714	3.833	0.079	0.20
300	0.75	0.1	0.000	2.917	3.833	1.517	0.35
300	0.75	0.2	0.000	3.269	3.833	1.134	0.05
300	0.75	0.5	3.090	3.623	3.833	0.241	0.20
500	0.25	0.1	0.000	3.589	3.833	0.847	0.55
500	0.25	0.2	3.705	3.814	3.833	0.047	0.85
500	0.25	0.5	3.705	3.763	3.833	0.065	0.45

Continued on next page

Table 1 – continued from previous page

Bees	Scouts	Top	Min	M	Max	SD	Percent
500	0.50	0.1	0.000	3.550	3.833	0.860	0.65
500	0.50	0.2	0.000	3.590	3.833	0.847	0.55
500	0.50	0.5	3.090	3.668	3.833	0.223	0.35
500	0.75	0.1	0.000	3.571	3.833	0.851	0.55
500	0.75	0.2	3.544	3.721	3.833	0.074	0.20
500	0.75	0.5	3.090	3.647	3.833	0.213	0.25
800	0.25	0.1	3.090	3.783	3.833	0.168	0.85
800	0.25	0.2	3.090	3.796	3.833	0.166	0.95
800	0.25	0.5	3.680	3.792	3.833	0.059	0.65
800	0.50	0.1	3.090	3.777	3.833	0.168	0.80
800	0.50	0.2	3.705	3.814	3.833	0.047	0.85
800	0.50	0.5	3.258	3.720	3.833	0.144	0.40
800	0.75	0.1	3.090	3.764	3.833	0.168	0.70
800	0.75	0.2	3.090	3.725	3.833	0.163	0.40
800	0.75	0.5	3.090	3.602	3.833	0.292	0.35
1000	0.25	0.1	3.090	3.767	3.833	0.179	0.80
1000	0.25	0.2	3.090	3.752	3.833	0.228	0.85
1000	0.25	0.5	3.090	3.764	3.833	0.168	0.70
1000	0.50	0.1	3.090	3.724	3.833	0.252	0.80
1000	0.50	0.2	3.090	3.776	3.833	0.169	0.80
1000	0.50	0.5	3.705	3.784	3.833	0.062	0.60
1000	0.75	0.1	3.090	3.758	3.833	0.168	0.65
1000	0.75	0.2	3.705	3.790	3.833	0.060	0.65
1000	0.75	0.5	3.537	3.769	3.833	0.090	0.60

Note. The table shows the aggregated results of the systematic variation across 20 seeds. Scouts and Top are given as fraction. Values in Min, Max, M und SD refer to the overall nectar level. Percent indicates how often the best solution was found given the specific hyperparameter settings.

Table 2
ANOVA for the Results of the Hyperparameter Variation

	Sum Sq	*df*	*F*	*p*
(Intercept)	185.84	1	1053.42	$< .001$
bees	56.44	5	63.99	$< .001$
percent_scouts	9.80	2	27.78	$< .001$
percent_top	3.87	2	10.96	$< .001$
bees:percent_scouts	0.99	10	0.56	.848
bees:percent_top	3.22	10	1.83	.052
percent_scouts:percent_top	4.53	4	6.41	$< .001$
bees:percent_scouts:percent_top	2.32	20	0.66	.870
Residuals	181.00	1026		

Table 3

Standardized factor loadings of the best BSO solution for the HS data set

	λ_g	$\lambda_{spatial}$	λ_{verbal}	λ_{mental}	λ_{memory}
visual	.602	.356			
cubes	.378	.294			
paper	.341	.374			
flags	.463	.455			
general	.521		.660		
paragrap	.581		.576		
sentence	.531		.699		
wordc	.567		.486		
wordm	.623		.579		
addition	.190			.747	
code	.439			.491	
counting	.311			.571	
straight	.441			.416	
wordr	.315				.535
numberr	.216				.595
figurer	.528				.337
object	.254				.494
numberf	.335				.371
figurew	.458				
deduct	.605				
numeric	.596			.270	
problemr	.676				
series	.746				
arithmet	.597			.273	

Note. $\chi^2(232, N = 301) = 414.6$, $p < .01$; Comparative Fit Index (CFI) = .933; Root Mean Square Error of Approximation (RMSEA) = .051 [.043; .059]. Standardized Root Mean Square Residual (SRMR) = .057.

Table 4

Standardized factor loadings of the best BSO solution for the SD3 data set

	λ_g	λ_M	λ_N	λ_P
M1	.421	.496		
M2	.770			
M3	.650		.139	
M4	.376	.204		
M5	.777	.165		
M6	.720	.190		
M7	.449	.531		
M8	.671			
M9	.632	.128		
N1	.298		.498	
N2	.149		.550	
N3	.474		.453	
N4	.396		.454	
N5	.524		.331	
N6	.246		.449	
N7	.368		.437	
N8	.354		.394	
N9	.560		.251	
P1	.624			.245
P2	.276			.485
P3	.559			.153
P4	.385			.415
P5	.583			.133
P6	.688			.160
P7	.138			.507
P8	.402			.310
P9	.743			.108

Note. M = machiavellianism, N = narcissism, P = psychopathy; $\chi^2(299, N = 18{,}192) = 17{,}073.8$, $p < .01$; Comparative Fit Index (CFI) = .902; Root Mean Square Error of Approximation (RMSEA) = .056 [.056; .057]. Standardized Root Mean Square Residual (SRMR) = .044.
